# SNP high-throughput screening in grapevine using the SNPlex™ genotyping system

**DOI:** 10.1186/1471-2229-8-12

**Published:** 2008-01-28

**Authors:** Massimo Pindo, Silvia Vezzulli, Giuseppina Coppola, Dustin A Cartwright, Andrey Zharkikh, Riccardo Velasco, Michela Troggio

**Affiliations:** 1 IASMA Research Center, Via E. Mach, 1 – I 38010 – San Michele all'Adige, Italy; 2 Myriad Genetics Inc, Wakara Way, 320 – UT 84108 – Salt Lake City, USA

## Abstract

**Background:**

Until recently, only a small number of low- and mid-throughput methods have been used for single nucleotide polymorphism (SNP) discovery and genotyping in grapevine (*Vitis vinifera *L.). However, following completion of the sequence of the highly heterozygous genome of Pinot Noir, it has been possible to identify millions of electronic SNPs (eSNPs) thus providing a valuable source for high-throughput genotyping methods.

**Results:**

Herein we report the first application of the SNPlex™ genotyping system in grapevine aiming at the anchoring of an eukaryotic genome. This approach combines robust SNP detection with automated assay readout and data analysis. 813 candidate eSNPs were developed from non-repetitive contigs of the assembled genome of Pinot Noir and tested in 90 progeny of Syrah × Pinot Noir cross. 563 new SNP-based markers were obtained and mapped. The efficiency rate of 69% was enhanced to 80% when multiple displacement amplification (MDA) methods were used for preparation of genomic DNA for the SNPlex assay.

**Conclusion:**

Unlike other SNP genotyping methods used to investigate thousands of SNPs in a few genotypes, or a few SNPs in around a thousand genotypes, the SNPlex genotyping system represents a good compromise to investigate several hundred SNPs in a hundred or more samples simultaneously. Therefore, the use of the SNPlex assay, coupled with whole genome amplification (WGA), is a good solution for future applications in well-equipped laboratories.

## Background

In the last few years, single nucleotide polymorphisms (SNPs) have become the most popular genetic marker system in both animals and plants. Their extraordinary abundance discovered in several genome sequencing projects [1], combined with recent technological improvements, makes SNP markers attractive for high-throughput use in marker-assisted breeding, EST mapping and the integration of genetic and physical maps.

At present several SNP identification methods are available such as resequencing of PCR amplicons with or without pre-screening, electronic SNP (eSNP) discovery in expressed sequence tag (EST) and shotgun genomic libraries. In these latter cases, sequences may be computationally screened for polymorphisms to distinguish true polymorphisms from sequencing errors if sufficient redundancy is present [2].

Unlike the first generation molecular markers, such as RFLPs (Restriction Fragment Length Polymorphisms) and RAPDs (Random Amplified Polymorphic DNAs), SNPs can be detected through non-gel-based high-throughput assays, saving both time and money [3]. Several SNP assay technologies have been developed based on various methods of allelic discrimination and detection platforms. Allele-specific hybridization, primer extension, oligonucleotide ligation and invasive cleavage represent four principal allelic discrimination reactions that can be coupled with several detection methods such as fluorescence, luminescence and mass measurements (see [4-6] for recent reviews). Recently, significant efforts towards large-scale SNP characterisation have been attempted in animals and plants with BeadArray technology (Illumina [7]) and the SNPlex™ genotyping system (Applied Biosystems Inc., ABI [8]). The selection of an appropriate genotyping method depends on many factors including cost, potential for multiplexing and throughput, equipment, and difficulty of assay development.

Until recently, only a few low- and mid-throughput methods have been used for SNP discovery and genotyping in grapevine (*Vitis vinifera *L.) [9-12]. The sequencing of the highly heterozygous genome of Pinot Noir [13], clone ENTAV 115, made it possible to identify millions of eSNPs as a potential source for high-throughput genotyping methods. Herein we report a successful application of the SNPlex genotyping system, which provided 563 new SNP-based markers anchoring the grapevine genome for future applied research programs.

## Results

### SNPlex and data analysis on genomic DNA (gDNA)

Of 949 candidate eSNPs selected from non-repetitive genome contigs, 813 passed the design rules of the SNPlex assay-design pipeline and were tested in 90 F_1 _progeny of Syrah × Pinot Noir (ENTAV 115) cross and in the two parental genotypes. 734 eSNPs passed the quality value using the rule-based method, with a mean of 5 failed SNPs and a 98% call rate per SNPset, while the remaining 79 were discarded from further analyses (Table 1). Of the 734 eSNPs, 171 were false positives. Of the remaining 563 eSNPs (See Additional file 1: Table S1 for the list of SNP sequences, submitted to the National Center for Biotechnology Information SNP database [1]), 509 followed the 1:1 or 1:2:1 Mendelian segregation ratio based on the chi-square test, whereas 54 showed an unexpected segregation ratio. Within the latter class, there were 46 cases where one parent was heterozygous and three clusters were observed (instead of the expected 2 with a 1:1 segregation ratio) and 8 cases where both parents were heterozygous and four clusters were detected (instead of the expected 3 with a 1:2:1 segregation ratio).

**Table 1:** Summary of the SNPset analyzed on Syrah, Pinot Noir and 90 Syrah × Pinot Noir progeny.

SNPset	Total SNPs (No.)	Passed SNPs (No.)	Genotypes (No.)	Assay pass rate (%)	Average call rate (%)
w0607103605_0001	48	45	4140	94	97
w0610104437_0001	48	42	3864	88	96
w0610104437_0002	47	42	3864	89	96
w0610104437_0003	47	46	4232	98	99
w0610104437_0004	48	41	3772	85	96
w0610104437_0005	48	41	3772	85	98
w0610104644_0001	48	42	3864	88	99
w0610104644_0002	48	43	3956	90	99
w0610104644_0003	48	43	3956	90	96
w0610104644_0004	48	44	4048	92	98
w0610104644_0005	48	42	3864	88	98
w0610104644_0006	48	45	4140	94	96
w0610104644_0007	48	46	4232	96	99
w0610104644_0008	48	44	4048	92	97
w0611104858_0001	48	41	3772	85	98
w0611104858_0002	47	42	3864	89	99
w0611104858_0003	48	45	4140	94	99
	813	734	67528	90	98

### SNPlex and data analysis on whole genome amplification DNA (WGA-DNA)

A total of 144 eSNPs combined in three 48-plex SNPsets (w0607103605_0001, w0610104437_0005 and w0611104858_0001) were also tested on WGA-DNA of the same 90 individuals. In this set, 15 eSNPs that did not pass the quality value on gDNA analysis were recovered on the WGA-DNA test, whereas three eSNPs that passed the quality value on gDNA failed on WGA-DNA (Table 2). Thirteen eSNPs failed both gDNA and WGA-DNA tests. The genotyping data were thoroughly consistent between the two analyses.

**Table 2:** Comparison between gDNA and WGA-DNA SNPlex genotyping assay

	gDNA	WGA-DNA
Number of genotypes	92	92
Number of SNPset	3	3
Number of eSNPs	144	144
Failed assay	17	3
Passed assay	102	116
Average call rate	98%	98%
Number of successful genotypes	9,384	10,672
Efficiency rate	70.8%	80.5%

### Resequencing analysis

**Figure 1:**
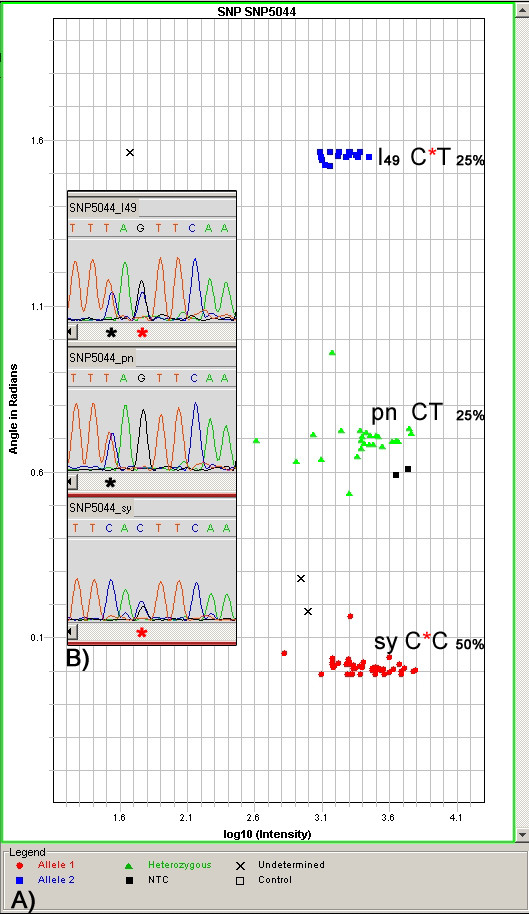
**SNP5044 polar cluster plot**. **A) **Genotype plot created by GeneMapper software v. 4.0 (ABI). The software analyzes the electropherogram and uses a clustering algorithm to assign the correct genotypes. SNP genotypes are displayed as a polar plot in which the intensity of the peaks is measured on the x axis and the ratio of both peak heights is measured on the y axis. **B) **Sequences flanking the target SNP5044 in Syrah (sy, bottom), Pinot Noir (pn, center) and one progeny (I_49_, top). The red asterisk indicates the unexpected additional polymorphism in Syrah and I_49 _near the target SNP represented by dark asterisk. The presence of the unexpected polymorphism explains the artificial homozygous cluster (C*T) shown in part A.

To validate the data obtained with the SNPlex assay, six regions containing SNP4165, SNP4057, SNP4045, SNP0102, SNP0054 and SNP5044 were resequenced in Syrah, Pinot Noir and six progeny. Four of them, SNP4165, SNP4057, SNP4045 and SNP0102, showed a Mendelian segregation and resequencing the corresponding regions confirmed the data obtained with the SNPlex analysis. For SNP5044 and SNP0054, which presented an additional homozygous cluster, an unexpected SNP was found within 10 bp from the target SNP in Syrah and in the progeny belonging to the additional homozygous cluster (Figure 1).

## Discussion

In this work, we report the first SNPlex genotyping system application in higher plants, which allowed the development of markers anchoring the grapevine genome. To date, a few low- and mid-throughput methods based on SSCP and minisequencing assay have been used for SNP genotyping in grape [9,10,12].

SNP markers have also been developed based on eSNP discovery in a 6.5× shotgun sequencing coverage of grapevine genome [13]. The efficiency rate of 69% with an average call rate of 98% exceeded the level recently achieved by resequencing selected ESTs (38.3%) and BAC-end sequences (35%) in previous study [11]. Although the efficiency was greatly enhanced, we observed that there were 10% systematic failed assays already detected in other SNPlex genotyping study (D. Sondervan *et al*., A. Kahler *et al*. and S. Bevan *et al*., SNPlex user meeting 2007) and 21% false polymorphisms. This unexpectedly high rate of false polymorphisms might be due to the SNPlex probe design based on a preliminary stage of contig assembly where the coverage of each haplotype in some regions was not sufficient.

In the WGA-DNA pilot study the genotyping data obtained from the gDNA and WGA-DNA analyses were thoroughly consistent, confirming previous SNP genotyping studies based on BeadArray [14,15] and Affimetrix technologies [16]. Moreover, the number of systematic failed assays was greatly reduced, from 10% to 2%, enhancing the average efficiency rate from 69% to 80%. These results were expected since MDA methods [17] provide a large amount of pure DNA with a uniform concentration among samples [18], meeting two basic requirements for a successful SNPlex assay. Resequencing of six SNP regions on WGA-DNA confirmed the SNPlex genotyping data and demonstrated the absence of amplification bias, as previously reported [16,17].

Resequencing of the SNP0054 and SNP5044 regions, belonging to the small group of SNPs with a distorted segregation, showed an unexpected additional polymorphism within 10 bp adjacent to the target eSNP in the Syrah genotype. Preferential ligation of one allele during probe annealing could explain the occurrence of an artificial homozygous cluster.

A large number of SNP genotyping technologies have been developed in the last few years. Different aspects, such as accuracy, reproducibility and level of throughput, should be taken into account when defining the most suitable SNP assay for breeding purposes. Moreover, flexibility, time and cost-effectiveness should be also considered, and in this regard, the turnaround time of the SNPlex analysis using a 3730xl DNA Analyzer (ABI) was about 30 min per sample. Thus 221,184 genotypes can be theoretically generated per day (48 runs/24 hours × 96 capillaries × 48-plex reaction).

## Conclusion

Unlike other SNP genotyping methods used to investigate either thousands of SNPs in few genotypes (i.e. BeadArray and Affymetrix technologies), or few SNPs in thousands of genotypes (i.e. TaqMan assay), the SNPlex genotyping system represents a good compromise to investigate several hundred SNPs in a hundred or more samples at the same time. Therefore, the use of the SNPlex assay, coupled with a WGA-DNA, is a good solution for medium- to large-scale genotyping studies in well-equipped laboratories.

## Methods

### Plant material and genomic DNA preparation

Genomic DNA of 90 F_1 _Syrah × Pinot Noir progeny and the two parental genotypes was isolated from 50–100 mg of young leaves. After freeze-drying, the leaf material was ground using the MM 300 Mixer Mill (Retsch Inc., Haan, Germany) and DNA extraction was performed using the DNeasy 96 Plant Mini Kit (Qiagen, Valencia, California, USA) according to the manufacturer's protocol.

### WGA

Ten ng of gDNA was amplified by MDA [17,19] using the GenomiPhi V2 DNA Amplification Kit (GE Healthcare, Little Chalfont, Buckinghamshire, United Kingdom) according to the manufacturer's protocol. The success of the MDA reaction and the absence of product in the negative control samples were assessed by agarose gel electrophoresis.

### SNP identification

The 6.5× shotgun sequence of Pinot Noir was the starting point of eSNP discovery. Approximately 6.2 million reads were produced by Sanger sequencing from 43 libraries with inserts of different sizes and assembled into contigs [13]. About 2.0 million SNPs were identified during the whole-genome shotgun assembly of Pinot Noir. Out of these, 949 SNPs, well-scattered along the 19 grape chromosomes were selected from non-repetitive contigs.

### Assay design

Allele-specific probes and optimized multiplexed assays using the SNPs of interest were designed by an automated multi-step pipeline [20]. These steps include: (1) entering the sequence containing target SNPs; (2) checking for formatting errors such as non-target polymorphisms near the target SNP or sequence motifs incompatible with the assay; (3) submitting the SNPs that passed the format check for the assay design. The ABI probe design prevents self-complementarity and dimerization, and annealing efficiencies are optimized for ligation. Furthermore, the optimal combination of SNPs to produce the highest yield per multiplex reaction is determined.

### SNPlex assay and data analysis

SNPlex was carried out on fragmented gDNA at a final concentration ranging from 45 to 225 ng and a final volume of 12.5 μl. Seventeen (fourteen 48-plex and three 47-plex) SNPset were analysed; of these, three SNPset (w0607103605_0001; w0610104437_0005 and w0611104858_0001) were also tested on fragmented GenomiPhi amplified gDNA (WGA-DNA) according to the manufacturer's protocol. The protocol was modified for the amount of PCR product used in the hybridisation cycles (3 instead of 1.5 μl).

Samples were run on the 3730xl DNA Analyzer (ABI) and data were analyzed using Gene Mapper v. 4.0 software (ABI). Genotype analysis was performed based on the SNPlex_Rules_3730 method following the factory default rules.

### Resequencing analysis

PCR primers were designed using the Primer3 software [21] according to the following criteria: 1) expected size of the amplified fragments between 200 and 600 bp; 2) primer size between 18 and 25 bases; 3) primer melting temperature (Tm) between 59 and 61°C; 4) alignment score and global alignment score for self-complementarity and complementarity between primer pairs ranging from 8 to 13.

Subsequently, six regions containing SNP4165, SNP4057, SNP4045, SNP0102, SNP0054 and SNP5044 were amplified in Syrah, Pinot Noir, and six progeny (I_49_, I_53_, I_56_, I_57_, I_58 _and I_59_) using templates in WGA-DNA. PCR reactions were performed using the following conditions: 1–20 ng of DNA template, 1× PCR buffer (Qiagen, Valencia, California, USA), 0.2 mM each dNTP, 0.4 μM of each primer, 1 U HotStarTaq DNA polymerase (Qiagen, Valencia, California, USA), and water to a final volume of 12.5 μl. DNA amplifications were performed using a 15 min initial denaturation/activation step, followed by 30 cycles at 94°C for 30 sec, 57°C for 30 sec, and 72°C for 2 min, with a final extension step of 10 min at 72°C. The PCR products were assessed by electrophoresis in 1.5% agarose gels and visualized by ethidium bromide staining. In order to remove unincorporated dNTPs and primers during the amplification reaction, 1 μl of exonuclease-phosphatase (ExoSAP-IT, GE Healthcare, Little Chalfont, Buckinghamshire, United Kingdom) was added to 1 μl of PCR product in a final volume of 6 μl and incubated at 37°C for 45 min followed by 72°C for 15 min.

The PCR product sequencing was carried out in both directions using the BigDye Terminator Cycle Sequencing Ready Reaction Kit v3.1 (ABI) as follows: 6 μl of PCR purified products, 5× Sequencing buffer, 0.32 μM of primer and 1 μl of BigDye Terminator in a final volume of 10 μl. Sequencing reactions were performed using a 2 min initial denaturation step, followed by 40 cycles at 96°C for 10 sec, 50°C for 5 sec and 60°C for 4 min. Prior to ethanol purification, capillary electrophoresis of PCR products was performed on a 3730xl DNA Analyzer (ABI). The DNA sequence electropherograms were aligned with the Pregap4/Gap4 software package (Staden Package, [22]) and used to survey parental alleles for polymorphic sites.

## Authors' contributions

MP carried out the SNPlex assay and data analysis, resequencing and drafted the manuscript. SV contributed to the SNPlex analysis and to discussion of the results. GC carried out the genomic DNA extraction and the MDA sample preparation. DC participated in the designing of the SNPlex assay. AZ carried out the genome assembly and SNP discovery. RV conceptualised the project and contributed to the discussion of the results. MT supervised the SNP-based marker development and genetic mapping and contributed to the discussion of the results. All authors read and approved the final manuscript.

## Supplementary Material

Additional file 1 **Table S1**. SNP IASMA ID number, NCBI SNP (ss) IDs, SNP allele, 5' near sequence allele and 3' near sequence allele respectively of the 563 SNP-based markers. Click here for file
